# Current Status of Soil-Transmitted Helminths among School Children in Kakamega County, Western Kenya

**DOI:** 10.1155/2016/7680124

**Published:** 2016-07-20

**Authors:** Teresia Ngonjo, Collins Okoyo, Julius Andove, Elses Simiyu, Agola Eric Lelo, Ephantus Kabiru, Jimmy Kihara, Charles Mwandawiro

**Affiliations:** ^1^Karatina University, P.O. Box 1957-10101, Karatina, Kenya; ^2^Kenya Medical Research Institute (KEMRI), Eastern and Southern Africa Centre of International Parasite Control, P.O. Box 54840-00200, Nairobi, Kenya; ^3^Technical University of Kenya (TUK), P.O. Box 52428-00200, Nairobi, Kenya; ^4^Kenyatta University, P.O. Box 43844-00202, Nairobi, Kenya; ^5^Division of Vector Borne Diseases, Ministry of Health, P.O. Box 20750-00202, Nairobi, Kenya

## Abstract

*Background.* School age children are at high risk of soil-transmitted helminth (STH) worldwide. In Kenya, STH infections in children remain high despite the periodic administration of anthelmintic drugs. Our study assessed the prevalence and intensity of STH in primary school-aged children in Kakamega County, western Kenya.* Methodology.* We carried out a cross-sectional study on a population of 731 children attending 7 primary schools in March 2014. Children aged 4–16 years were examined for STH by the quantitative Kato-Katz technique. Infection intensities were expressed as eggs per gram (epg) of faeces.* Findings.* Among 731 school children examined for STH, 44.05% were infected. Highest prevalence of STH was in Shitaho primary school where 107 participants were examined and 62.6% were infected with mean intensity of 11667 epg. Iyenga had the least prevalence where 101 participants were examined and 26.7% were infected with mean intensity of 11772 epg.* A. lumbricoides* was the most prevalent STH species with 43.5% infected, while hookworm infections were low with 1.8% infected.* Conclusion.* Prevalence of STHs infections in Kakamega County remains high. We recommend guidelines and other control strategies to be scaled up to break transmission cycles.

## 1. Introduction

Soil-transmitted helminths (STHs) infections represent a major public health problem in poor and developing countries and have constituted a universal burden which does depend not only on regional ecological condition but also on local standard of social and economic development of the people [1]. Many different species of soil-transmitted helminths infect humans, especially in the tropical and subtropical parts of the developing world [2]. Four nematodes, in hundreds of millions of human infections, in particular stand out because of their widespread prevalence. These include roundworm,* Ascaris lumbricoides*, the whipworm* Trichuris trichiura*, and two species of hookworm,* Necator americanus* and* Ancylostoma duodenale* [2].

Today, it is estimated that approximately one-third of the almost three billion people that live on less than two US dollars per day in developing regions of Sub-Saharan Africa, Asia, and the Americas are infected with one or more helminths [3, 4]. The most common helminthiases are those caused by intestinal helminths, ascariasis, trichuriasis, and hookworm. This means that the inhabitants of thousands of rural, impoverished villages throughout the tropics and subtropics are often chronically infected with several different species of parasitic worm; that is, they are polyparasitized [3, 4].

For reasons not well understood, compared with any other age group, school-aged children (including adolescents) and preschool children tend to harbor the greatest numbers of intestinal worms and as a result experience growth stunting and diminished physical fitness as well as impaired memory and cognition [5]. These adverse health consequences combine to impair childhood educational performance, reduce school attendance [6], and account for the observation that hookworm and presumably other diseases caused by parasitic worms reduce future wage-earning capacity [7].

Global commitment focuses on school based chemotherapy programmes to implement helminth control strategies [8]. Currently it is estimated that more than 2 billion people worldwide are infected with STH. As discussed elsewhere, the majority of this population resides in low and middle income countries (LMICs) [7, 8]. Together with LMICs, STH endemicity is concentrated in areas where ecological and environmental characteristics facilitate transmission [8, 9].

Global numbers infected with STH infection will remain an elusive goal, due in part to a scarcity of reliable and accurate epidemiological data and in part lack of specificity of clinical signs due to STH [10, 11]. Ministry of Health's Department of Child Health in Kenya seeks to promote good health and nutrition. The ministry recognizes the detrimental effects of STH infections in primary-school-aged children [12]. School children serve in assessment of helminths distribution and prevalence. School based deworming programmes provide information and help to design implementation of cost-effective control programme that aims to monitor STH impact at both national and regional levels. The objective of this cross-sectional study was to determine the prevalence of soil-transmitted helminths infections among school children in informal urban settlements in Kakamega, Western Kenya, prior to a National Deworming Mass Drug Administration.

## 2. Materials and Methods

### 2.1. Study Site

The study was carried out in Kakamega County. It is located at 00°20′N 34°46′E with an area of 3,033.8 km. The climate is mainly tropical, with variations due to altitude. Kakamega County is mainly cool and wet most of the year. The county has a mixture of both subsistence and cash crop farming, with sugar cane being the preferred large-scale cash crop. It has a population of 1,660,651 people [13].

### 2.2. Study Population

Pupils in early childhood, classes two to six from Kakamega East, South, and Central Sub-Counties, formed the study group. Pupils from schools that have not been dewormed in the last year and those who have been dewormed six months in the National Deworming Programme prior to the commencement of the study were included. Those who have been dewormed within the three months were not included. Children below 4 years and above 16 years were not included in the study. A list of registered pupils in each class was obtained from the head teacher in each school and age of each participating pupil was confirmed from parent when obtaining the informed consent. Parents and guardians consented to have the results of the study published while adhering to the privacy of the study subjects.

### 2.3. Study Design and Sampling

Seven schools were randomly selected in Kakamega County. A total of 731 children in 7 primary schools in 3 sub-counties of Kakamega County were recruited in the study. At each selected school, 9 boys and 9 girls were randomly selected from classes 2 to 6 and early childhood to have a total of 108 children per school. This was a cross-sectional study to assess the prevalence and intensity among the sampled children before mass drug administration (MDA) in school-aged children in the study area.

### 2.4. Parasitological Examination

Each participating child was asked to provide fresh stool sample for Kato-Katz examination to detect soil-transmitted helminths infections. Each pupil was guided on how to provide about 2 grams of stool samples. Diagnosis of helminths was determined by presence of egg counts of soil-transmitted helminths (STHs) in defined quantities of stool using Kato-Katz technique [14]. Two Kato slides per stool sample were prepared using fixed quantity of sieved 41.7 mg (WHO kit) of stool on a punched template. It was then mounted on slides and covered with malachite green impregnated cellophane. The slides were observed within one hour under the microscope at a magnification of ×10. For hookworms, the slides were read immediately while* Ascaris lumbricoides and Trichuris trichiura* eggs were examined 60 minutes later. The total numbers of eggs were expressed as eggs per gram (epg) of faeces. Quality control was performed by systematic random examination, by the team leader of 10% of the daily examined Kato-Katz slides.

### 2.5. Statistical Analysis

Observed prevalence of each STH species was calculated at school, sub-county, and regional levels, and 95% confidence intervals (95% CIs) were obtained by binomial logistic regression, taking into account clustering by schools. Comparisons of prevalence by location, age group, and sex were tested for significance on the basis of the Wald test. For the purposes of analysis, the following age groups were used: 3–5, 6-7, 8-9, 10-11, 12-13, and above 13 years old. Mean egg counts were expressed as arithmetic mean epg and since egg counts are not normally distributed, 95% CIs were estimated using a negative binomial regression model. Infection intensities were classified into light to heavy intensity of infections according to WHO guidelines [15], and the prevalence of light to heavy infections and 95% CIs adjusted for school clusters were also obtained using binomial regression.

## 3. Results

**Figure 1:**
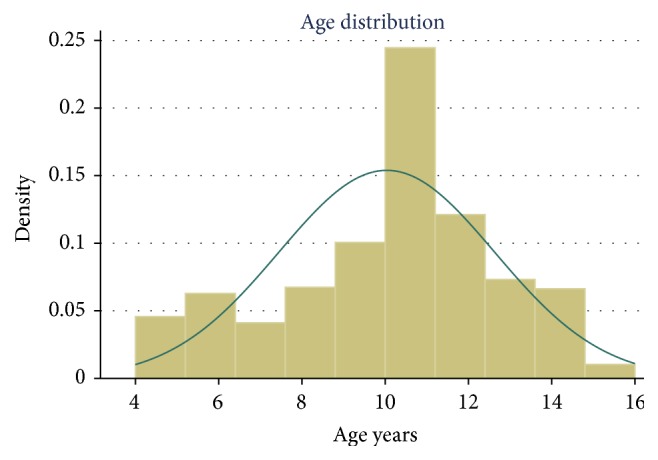
The age distribution of the children sampled for the study.

Overall, data was collected from 731 children in 7 primary schools in 3 sub-counties of Kakamega County. Of 731 participants, 28.5% of the pupils were from Kakamega South Sub-County, 43.1.% from Kakamega East Sub-County, and 28.9% from Kakamega Central Sub-County. The children had almost equal representation in terms of gender. Of the 731 participants examined, 50.2% were males and 49.8% females. The mean age of pupils was 10 years (SD = 2.6 years) with age range of 4 years to 16 years (Figure 1).

### 3.1. Overall Prevalence and Intensity

Of the 731 participants examined for STHs combined, 44.05% (95% CI: 35.80–54.20) were infected.* A. lumbricoides* was the most prevalent STH species. Out of the total participants, 43.5%, (95% CI: 35.21–53.74) were infected. Hookworm and* T. trichiura* had generally low prevalence. Of those examined, 0.27%, (95% CI: 0.06–1.09) and 0.82%, (95% CI: 0.38–1.78) were infected, respectively (Table 1).

The overall mean intensity of the combined STH species was 36743 epg, (95% CI: 2560–5274).* A. lumbricoides* had the highest mean intensity of 3673 epg, (95% CI: 2559–5274); hookworm and* T. trichiura* had generally low mean intensity of 0.33 epg, (95% CI: 0.05–2.29) and 0.84 epg, (95% CI: 0.16–4.38), respectively (Table 1).

### 3.2. Soil-Transmitted Helminths (STHs) Distribution within Schools and Sub-County

The prevalence and mean intensity for any STHs species were examined for surveyed schools, sub-county, gender, class, and age groups taking into account clustering by schools.

Overall, among the 7 schools surveyed, Shitaho primary school had the highest prevalence of STHs combined. Of the 107 participants examined, 62.6% (95% CI: 54.1–72.5) were infected with mean intensity 11667 epg (95% CI: 8400–16205). Bukusi primary school had 99 participants; 55.6%, (95% CI: 46.6–66.3) were infected with mean intensity 7368 epg (95% CI: 4827–11246). Iyenga primary had the least prevalence. Of the 101 participants examined, 26.7% (95% CI: 19.4–36.9) were infected with mean intensity of 11772 epg (95% CI: 6889–20115) (Table 2).

Hookworm was only observed in Bukhulanya primary school. Of the 110 participants in that school, 1.8% (95% CI: 0.5–7.2) were infected with mean intensity of 120 epg (95% CI: 41–355). Kakamega Central had the highest prevalence of STHs combined. Of the 211 participants examined, 52.1% (95% CI: 34.9–77.8) were infected with mean intensity of 10768 epg (95% CI: 8822–13144). In the same sub-county, 52.1% (95% CI: 35–77.8) were infected with* Ascaris lumbricoides* with mean intensity of 10763 epg (95% CI: 8808–13153). Only 0.9%, (95% CI: 0.1–6.9) of the participants examined in Kakamega Central were infected with* T. trichiura* with mean intensity 264 epg (95% CI: 39–1804). However, hookworm was only prevalent in Kakamega South district. Of the 208 participants examined, 0.9% (95% CI: (0.1–6.2) were infected with hookworm with mean intensity of 120 epg (95% CI: 41–355) (Table 3).

### 3.3. Soil-Transmitted Helminths (STHs) Distribution within Gender and Age Categories

**Figure 2:**
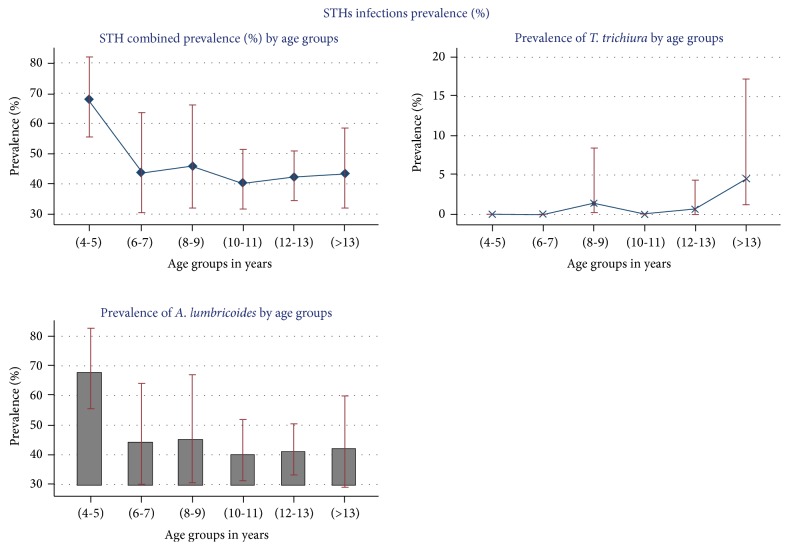
Prevalence (%) of various STHs species by age category.

The prevalence of STH combined infection and any single STHs did not vary markedly between males and females participants (Table 4). Generally, children with 4-5 years showed highest prevalence. Of the total examined, 67.5% (95% CI: 55.5–82.1) were infected for STH combined and* A. lumbricoides* species compared to other age groups. However, low levels of hookworm prevalence were present among (6-7) years and (12-13) years (Figure 2).

**Figure 3:**
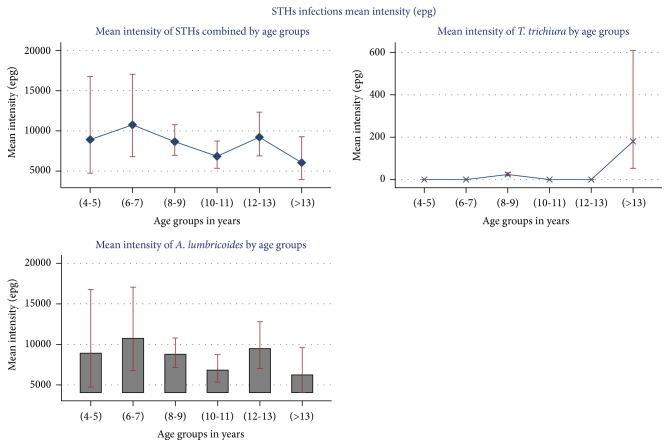
Mean intensity (epg) of various STHs species by age category.

Children with age (4-5) years had the highest prevalence of STHs combined (67.5%) compared to other age groups, whereas the mean intensity of STHs combined was high in the age groups (6-7) years and (12-13) years, 10,752 epg and 9,217 epg, respectively (Figure 3).

Similarly, prevalence of* A. lumbricoides* was highest among pupils of (4-5) years, 67.5% with other age categories showing prevalence ranging between 45% and 40%. Generally, there were high levels of* A. lumbricoides* mean intensity in age groups (6-7) and (12-13) years. Children above 13 years had the least mean intensity of* A. lumbricoides*, 4,241 epg (Figure 3). Low levels of* T. trichiura* prevalence were only found in age groups (8-9), (12-13), and (>13) while the remaining age groups had no* T. trichiura* infection. Low levels of mean intensity of infection were only recorded in age groups (8-9) and (>13) years (Figure 3).

### 3.4. Light-Heavy Intensity of Intestinal Helminths

Intestinal infections were categorized from light-heavy intensities according to WHO guidelines [15]; however, only* A. lumbricoides* intensity could fit into more than one category, while the remaining infections studied had light intensities. 80.6% of the pupils had light intensity of* A. lumbricoides* while 19.4% had moderate intensity.

## 4. Discussion

The study assessed the prevalence of three soil-transmitted helminths infections in school children in three sub-counties, namely, Kakamega Central, Kakamega South, and Kakamega East. The overall STHs infections had varied prevalence in the different schools and sub-counties. It was evident that Kakamega Central Sub-County had the highest prevalence (52.1%) of STHs infections. This was far much higher than what was reported in the neighbouring Sub-County Kisumu East, in Nyanza County (17.4%), and Kilindini in Mombasa County (18.0%) [16]. The most common STHs detected among children were* A. lumbricoides*, followed by very low prevalence (0.9%) of* T. trichiura*. However, hookworm was only reported in Kakamega South Sub-County with low levels of prevalence reported among children of ages (6-7) years and (12-13) years. This suggests low exposure to infective hookworm larvae that commonly occurs outside the household like in agricultural areas or in defined defecation sites [17]. There was low* T. trichiura* prevalence in Bukhulanya, Matende, Bukusi, and Shina primary schools. This showed that there is a remarkable decrement in the prevalence in some of parasitic infections. The progress in the reduction of the parasites prevalence could be due to different interventions that have been undertaken in the area like national school based deworming and health education. Differences between sites may be explained by the relative importance of domains in defining contact with infectious stages [18].

The prevalence of STHs combined and any single STHs did not vary markedly between males and females participants. These results agree with those from a study conducted in the central part of Turkey that showed there was no statistically significant difference observed between presence of intestinal prevalence and gender [19]. Overall, high prevalence and intensities were observed in age group 4-5 years (67.5%) compared to other age groups, whereas the mean intensity of STHs combined was high in the age groups (6-7) years and (12-13) years, 10,752 epg and 9,217 epg, respectively. These results were different from those of Kibera where 16.2% prevalence was reported among children aged 10–18 years living in urban slum settings in Nairobi Kenya [20].

Prevalence of* A. lumbricoides* was high among pupils of (4-5) years which was 67.5% with other age categories showing prevalence ranging between 45% and 40%. This prevalence was much higher than* A. lumbricoides* in Thika Sub-County, Kiambu County [21]. There were higher levels of* A. lumbricoides* mean intensity in age groups (6-7) and (12-13) years. Children above 13 years had the least mean intensity of* A. lumbricoides.* These results compare with a study carried out in Honduras where children aged 5 to 12 years had a higher percentage of* A. lumbricoides* infections than individuals in the other age groups. However, both the prevalence and intensity of* A. lumbricoides* varied significantly by age (*p* < 0.001).

The different prevalence of STHs in the study is in agreement with previous studies from Western Kenya, although higher percentage prevalence was noted for* A. lumbricoides* infections (43.5%) in this study. Current study had low prevalence hookworm and* T. trichiura*, 0.27% and 0.82, respectively. Informal settlements of Kisumu had prevalence of 4.9% for* A. lumbricoides* and 7.7% for* T. trichiura* [22]. In line with a recent STH survey in these regions,* A. lumbricoides* infections were more prevalent compared to* T. trichiura* and hookworm [23].

It was remarkable feature that this study showed that high numbers of children were infected with* A. lumbricoides*. It could be due to the fact that transmission of* A. lumbricoides* is through faecal oral route and infections after treatment reappear fast, particularly for* A. lumbricoides* [23]. Coinfections of STHs were not common in the study area. Multiple STH infections were less common than in other studies elsewhere in East Africa [16, 24]. In Busia, Kenya, 26% of children were infected with all 3 STHs and 31.1% with 2 STHs [16]. Current study also differs with what was observed in Pemba, Tanzania, where 67% of children were infected with all 3 STHs and 28% with 2 STHs [25]. Such differences might have arisen due to differences in the study subjects, sociodemographic conditions, and socioeconomic characteristics of the areas. Coinfection may affect nutritional status of the children because of the combined effect of the different parasites that can deprive the children of the important nutrients. Again children with a single infection stand out with a better academic score than those with multiple infections [26].

Our findings confirm that the children did not harbor multiple infections of STHs. This is an important observation and could be attributed to impact of the school based approach adopted control programme. Low prevalence and mean intensity of hookworm and* T. trichiura* in this study are an implication that mass treatment has reduced their refugia and the rate of infection was low. However, the prevalence of* A. lumbricoides* is significantly high. This is an endemic area where there is high daily occurrence of transmission because of soil and food contaminated with infective stages of this parasite [27]. This could lead to high morbidity in the children and predisposing factors for this parasite require to be improved.

The mean intensities in hookworm and* T. trichiura* were quite low and were all in light infection. This indicates less contamination of the environment with infective larvae. Hookworm has a slower rate of infection compared to* A. lumbricoides* because its third-stage larvae have shorter life expectancy (3–10 days) unlike* A. lumbricoides* eggs with several months' infective period [28]. This implies that the environment can recover more quickly from hookworm contamination than from* A. lumbricoides*. This could have been attributed to the high prevalence of* A. lumbricoides* and its moderate-light infection in the current study. Reduced prevalence of hookworm and* T. trichiura* could also be attributed to high community level access to improved sanitation, as well as county economy and health service delivery indicator scores. School based intervention should be established in order to reduce infection rates.

When comparing our baseline survey results to historical data, STH infection prevalence has strongly decreased over the last decade in certain regions of Kenya where a deworming programme has been implemented since 1998 [29, 30]. However, Kakamega County in Western Kenya has remained a high endemic area. Our study did not assess risk factors associated with STH. However, it is evident from our results that prevalences of STH were high in this area, an indication that transmission is high and could be influenced by conditions that determine the development and survival of free-living stages in the external environment [28]. Survival of STH free-living stages is dependent on environmental conditions that influence their transmission success. Limited access to water, sanitation, and hygienic behaviour determines the rate of exposure to ova and larvae [31].

## 5. Conclusion

The analysis of collected data provided useful insight into the current prevalence and mean intensity of soil-transmitted helminths (STHs) infections in Kakamega County.* A. lumbricoides* is the predominant STH in the region. We advocate for integrated, multisectoral approaches prioritizing communities where focal transmission shows singular patterns. Factors that facilitate STH transmission in this region and their impact on prevalence and intensity should be considered in future surveys.

## Figures and Tables

**Table 1:** Overall prevalence and mean intensity of STH infections.

Infection	Prevalence (%)	95% CI
STHs combined	44.05	(35.80–54.20)
Hookworm	0.27	(0.06–1.09)
*A. lumbricoides*	43.5	(35.21–53.74)
*T. trichiura*	0.82	(0.38–1.78)

Infection	Mean intensity (epg)	95% CI

STHs combined	3674	(2560–5274)
Hookworm	0.33	(0.05–2.29)
*A. lumbricoides*	3673	(2559–5274)
*T. trichiura*	0.84	(0.16–4.38)

**Table 2:** Prevalence and mean intensity of soil-transmitted helminths (STHs) per school.

	STHs combined		Hookworm		*A. lumbricoides*		*T. trichiura*	
	Pr% (95% CI)	Avg intensity	Pr% (95% CI)	Avg intensity	Pr% (95% CI)	Avg intensity	Pr% (95% CI)	Avg intensity
*School*								
*Bukhulanya*	34.5 (26.7–44.7)	6538 (4115–10387)	1.8 (0.5–7.2)	120 (41–355)	32.7 (25.0–42.8)	6893 (4364–10888)	1.8 (0.5–7.2)	24 (18–32)
*Bukusi*	55.6 (46.6–66.3)	7368 (4827–11246)	0	0	54.5 (45.6–65.3)	7504 (4927–11427)	1.0 (0.1–7.1)	0
*Iyenga*	26.7 (19.4–36.9)	11772 (6889–20115)	0	0	26.7 (19.4–36.9)	11772 (6889–20115)	0	0
*Matende*	41.3 (32.9–52.0)	9367 (6002–14621)	0	0	41.3 (32.9–52.0)	9355 (5937–14740)	1.9 (0.5–7.6)	264 (39–1804)
*Shamusinjiri*	39.3 (31.0–49.7)	5973 (3881–9194)	0	0	39.3 (31.0–49.7)	5973 (3881–9194)	0	0
*Shina*	48.5 (39.8–59.2)	5584 (3820–8161)	0	0	47.6 (38.8–58.3)	5697 (3929–8262)	1.0 (0.1–6.8)	0
*Shitaho*	62.6 (54.1–72.5)	11667 (8400–16205)	0	0	62.6 (54.1–72.5)	11667 (8400–16205)	0	0

**Table 3:** Prevalence and mean intensity of soil-transmitted helminths (STHs) per sub-county.

Sub-county	STHs combined		Hookworm		*A. lumbricoides*		*T. trichiura*	
	Pr% (95% CI)	Avg intensity	Pr% (95% CI)	Avg intensity	Pr% (95% CI)	Avg intensity	Pr% (95% CI)	Avg intensity
Kakamega South	44.7 (28.4–70.3)	7029 (6285–7860)	0.9 (0.1–62)	120 (41–355)	3.3 (26.6–70.4)	7259 (6707–7857)	1.4 (0.8–2.5)	24 (19–30)
Kakamega East	38.3 (27.8–52.6)	7125 (4594–11050)	0	0	37.9 (27.9–51.6)	7185 (4667–11064)	0.3 (0–2.3)	0
Kakamega Central	52.1 (34.9–77.8)	10768 (8822–13144)	0	0	52.1 (35–77.8)	10763 (8808–13153)	0.9 (0.1–6.9)	264 (39–1804)

**Table 4:** Prevalence and mean intensity of soil-transmitted helminths (STHs) per gender.

Gender	STHs combined		Hookworm		*A. lumbricoides*		*T. trichiura*	
	Pr% (95% CI)	Avg intensity	Pr% (95% CI)	Avg intensity	Pr% (95% CI)	Avg intensity	Pr% (95% CI)	Avg intensity
Male	44.1 (37.2–52.4)	7150 (5474–9339)	0.3 (0–1.8)	0	43.3 (36.4–51.5)	7281 (5647–9386)	1.4 (0.6–2.9)	118 (32–432)
Female	44.0 (32.6–59.3)	9549 (7250–12577)	0.3 (0–2.0)	0	43.7 (32.2–59.3)	9607 (7312–12624)	0.3 (0–2.0)	0
